# Characteristics of Disclosure of Suicidal and Nonsuicidal Behaviors in a Clinical Sample of Adolescents

**DOI:** 10.1007/s10802-024-01269-8

**Published:** 2024-11-26

**Authors:** Amy M. Brausch, Taylor Kalgren, Chelsea Howd

**Affiliations:** Department of Psychological Sciences, Western Kentucky University, 1906 College Heights Blvd, Bowling Green, KY 42101 USA

**Keywords:** Disclosure, Adolescents, Suicide ideation, Suicide attempt, Nonsuicidal self-injury, Reasons for living

## Abstract

Many adolescents fear disclosing self-injurious thoughts and behaviors (SITBs) due to stigma or concern about responses from others. The current study examined rates of disclosure for nonsuicidal self-injury (NSSI), suicide ideation, and suicide attempts in a clinical sample of adolescents, and identified the individuals to whom they disclosed their SITBs. Differences in reasons for living (parent and peer support, future optimism, self-acceptance, and fear of suicide) were examined across disclosure groups. The sample included 100 adolescent inpatients (mean age = 14.61). Rates of disclosure were relatively high: 77% for NSSI, 75% for suicide ideation, and 83.7% for suicide attempts. Adolescents who disclosed NSSI reported higher scores on subscales of self-esteem and future optimism compared to those who did not disclose. No differences were found for adolescents disclosing to parents vs. others; only the fear of suicide subscale was significantly different, and was lower for adolescents who disclosed NSSI to peers vs. others. Adolescents with suicide ideation disclosure reported more parent support compared to those who had not disclosed, those with peer disclosure reported lower fear of suicide than those disclosing to others, and there were no differences for disclosing to parents vs. others. For suicide attempts, only self-acceptance subscale scores were significantly different, and lower for adolescents who disclosed to peers vs. others. There were no differences for disclosing suicide attempts to parents versus other people. The willingness to disclose self-harm behaviors seems influenced by the perception of safety and anticipated support from parents or others to whom adolescents disclose.

Adolescents continue to experience self-injurious thoughts and behaviors (SITBs) at relatively high rates worldwide. Global lifetime prevalence of suicidal ideation in adolescents is reported to be between 14 and 23% and lifetime prevalence of suicide attempts is between 5 and 16% (Van Meter et al., 2023). While these numbers have been increasing annually, the prevalence of nonsuicidal self-injury (NSSI) in adolescents have also been high, with more than 17% reporting at least one act of NSSI within the past 12 months in the United States alone (Monto et al., 2018), and 22% reporting lifetime NSSI globally (Xiao et al., 2022). Rates of NSSI for adolescents have been on an upward trend, increasing 6.2% from 2020 to 2021 after the start of the COVID-19 pandemic (Zetterqvist et al., 2021). Although SITBs in adolescents are an increasing and alarming issue, many adolescents fear disclosing these experiences due to concerns about stigma, confidentiality breaches, or being labeled as attention-seeking (Waller et al., 2023). Clinicians often rely on disclosure to provide adequate treatment and safety planning. Support from parents, peers, or others also rely on disclosure. It has been demonstrated across multiple settings that the personal qualities of the individual to whom the disclosure is made, such as the ability to recognize signs of self-harm and comfortability, facilitate the disclosure of SITBs in adolescents (Waller et al., 2023). However, there is limited research on the personal qualities of the adolescent who is disclosing their SITBs, particularly in clinical samples in the United States. The current study examined rates of disclosure of NSSI, suicide ideation, and suicide attempts in a clinical sample of adolescents, and identified the individual(s) to whom they disclosed their SITBs. Additionally, we examined if disclosure was related to multiple reasons for living including parent and peer support, self-acceptance, future optimism, and fear of suicide.

## Disclosure in Adolescents

Previous research on adolescent SITBs reports various disclosure rates between types of self-harm (i.e., NSSI, suicide ideation, or suicide attempts) and the geographic location of the sample. Between 37 and 80% of adolescents who engage in NSSI report disclosing their behaviors to others with higher rates of disclosure being reported in samples from the United States (Berger et al., 2013; Demuthova et al., 2020; Fox et al., 2022; Hasking et al., 2015). Between 39 and 78% of adolescents who experience suicide ideation report disclosing their thoughts, again, with higher rates of disclosure being reported in the United States (Calear & Batterham, 2019; Eskin, 2003; Fox et al., 2022; Shin et al., 2023). There is less research on disclosure of suicide attempts in adolescents; however, one study found that 73% of adolescents reported disclosing their suicide attempts in the U.S. (Fox et al., 2022). A study of general self-harm behaviors (defined broadly as NSSI, suicide attempts, lost a job on purpose, driving recklessly, etc.) reported that 55% of adolescents disclosed these behaviors to **s**omeone (Demuthova et al., 2020). Notably, the majority (69–80%) of adolescents who disclosed any SITBs reported disclosing to a peer (Demuthova et al., 2020; Eskin, 2003; Hasking et al., 2015). These rates are well above those who report disclosing to parents or a mental health professional (27% and 10%, respectively; Demuthova et al., 2020; Eskin, 2003; Hasking et al., 2015). Additional research has revealed that adolescents are more inclined to disclose SITBs to same-sex friends or when discussing NSSI rather than suicidal thoughts and behaviors (Eskin, 2003; Fox et al., 2022). Eskin (2003) found that Swedish adolescents who did disclose their suicidal thoughts reported lower levels of suicide ideation compared to adolescents with similar thoughts who did not disclose. Additionally, research has been predominantly focused on disclosure of SITBs in community samples (Rowe et al., 2014; Simone & Hamza, 2020). Few studies in these reviews examined a clinical sample of adolescents, and none originated from the United States. Rates of SITB disclosure in clinical samples may differ from community samples as staff knowledge, training, and experience have been shown to facilitate disclosure of SITBs in young people under 18 years of age (Waller et al., 2023). Examining adolescent disclosure of SITBs in a clinical sample is especially important as rates of these behaviors are often higher compared to community samples (Robinson et al., 2023). Considering disclosure rates in clinical settings in the U.S. have yet to be examined, it is unclear how disclosure rates will compare to adolescents in community settings. It is also important to examine to whom adolescents in these settings disclosed their SITBs, as a reported barrier to disclosure is fear of hospitalization (Waller et al., 2023).

## Barriers to Disclosure

Previous research has examined why adolescents chose not to disclose their SITBs to others, revealing themes relating to internal emotions (e.g., shame or guilt) and their perception of others’ reactions. Several studies found that adolescents with SITBs often experience embarrassment or shame, hindering their help-seeking efforts (Fox et al., 2022; Rosenrot & Lewis, 2020). Moreover, these studies highlighted adolescents' concerns about the stigma of SITBs and negative reactions from their friends and family, including rejection and perceived burdensomeness. Levi-Belz and colleagues (2019) identified that interpersonal difficulties, particularly low levels of parental support, could significantly hinder adolescents' disclosure of suicide attempts. Additionally, adolescents with SITBs have expressed fears regarding breaches of confidentiality, worrying that their parents might be informed or that they might be subjected to hospitalization (Fox et al., 2022). Common barriers preventing the disclosure of suicidal ideation to mental health professionals included the belief that suicidal thoughts would go away, apprehensions about judgment or unsupportive responses from mental health professionals, and doubts about the efficacy of mental health interventions (McGillivray et al., 2022). Simone and Hamza (2020) noted similar barriers regarding disclosure of NSSI among adolescents and young adults, including the fear of rejection or harsh judgment, accusations of attention-seeking, and the broader societal stigma that could have adverse impacts on future opportunities. Research indicates that social support from family and peers may play a pivotal role in disclosure of SITBs among adolescents.

## Peer and Parent Support Roles in Disclosure

Adolescence marks a crucial developmental stage where peers wield considerable influence. Although seeking help from a formal source (e.g., counselor, psychiatrist) allows for optimal treatment and safety planning, research shows most adolescents disclose their SITBs to friends rather than mental health or medical professionals (Demuthova et al., 2020; Eskin, 2003; Hasking et al., 2015). Peer-to-peer models have been used in suicide prevention paradigms for years and are implemented in many different programs such as gatekeeper training and crisis support lines. The bulk of gatekeeper training research indicates that these training programs show promise in enhancing knowledge and attitudes toward suicidal intervention, ultimately aiding in stigma reduction, a recognized barrier to disclosure (Bowersox et al., 2021). While these programs show an increase in intrapersonal skills and can help peers with resources and knowledge for when adolescents eventually do disclose their SITBs (Samuolis et al., 2019), there is limited rigorous evidence relating to long-term changes in behavior from those who received training or overall effectiveness in suicide prevention (Bowersox et al., 2021). However, crisis lines allow peers to utilize these skills in a safe and confidential space for adolescents to disclose their behaviors during a crisis. Research on crisis lines shows effectiveness for adolescents via reduced depression, increased well-being, and reduced severity of suicidal ideation from pre- to post-test (Fukkink & Hermanns, 2009; King et al., 2003; Sindahl et al., 2019). Although effectiveness plays a crucial role in suicide prevention, research is needed to understand what influences adolescents’ decisions to reach out and disclose their self-harm behaviors.

Parents also play a unique role in SITB prevention during adolescence. Research examining adolescents' perspectives regarding the support they can receive after disclosing to their friends found that 12% of peers who were disclosure recipients suggested that the adolescent could tell an adult (i.e., parent, relative, or teacher; Berger et al., 2017). Although previous research indicates that adolescents are typically more inclined to share their SITBs with peers, a recent scoping review consistently identified parents as the primary confidants for suicide ideation disclosure, followed by friends and then mental health professionals (Davies et al., 2024). However, Hedeland and colleagues (2013) reported that among adolescents hospitalized after a suicide attempt, 62.5% had attempted to discuss their feelings with their parents beforehand but felt unheard. Similarly, Pisani and colleagues (2012) noted that 22.8% of high school students disclosed their suicidal ideation to an adult, with 67.1% doing so specifically to seek help. Notably, students were significantly more likely to disclose to an adult than a peer when they sought assistance, with disclosure rates ranging from two to five times higher.

Despite recognizing the potential benefits of disclosing to adults, research indicates that only around 10% of adolescents with SITBs share this information with their parents (Demuthova et al., 2020; Eskin, 2003; Hasking et al., 2015). Parents may initially downplay these thoughts and behaviors as attention-seeking behavior, delaying intervention and underestimating their seriousness (Oldershaw et al., 2008). However, many parents report regretting not intervening earlier, realizing it could have prevented further escalation (Oldershaw et al., 2008; Rissanen et al., 2009a). Concerningly, adolescents often perceive their parents' reactions to be either overreactive or dismissive (Rissanen et al., 2009b), and research generally reports low to moderate agreement between parents and their children regarding suicidal thoughts, with about 50% of parents unaware of their child's suicidal ideation (Jones et al., 2019). Furthermore, only 16–26% of adolescents with SITBs report that their parents know about their behavior (Berger et al., 2013), even though parent involvement is crucial for adolescent intervention for SITBs (Glenn et al., 2019). Mental health providers sometimes share information about SITBs to parents without adolescent consent, potentially hindering future disclosure to these professionals (Fox et al., 2022). To promote disclosure and support, parents can create a nurturing environment, as parental support is a known protective factor against NSSI in adolescents (Valencia-Agudo et al., 2018). Additionally, parental warmth and parent connectedness have been found to buffer the impact of suicide ideation and attempts in adolescence (Abdelraheem et al., 2019; Connor & Rueter, 2006; Kidd et al., 2006). Through promoting healthy peer and parent relationships, adolescents may feel more inclined to disclose their thoughts of SITBs, facilitating early intervention and support.

## Current Study

While some research has explored disclosure rates of SITBs among adolescents along with their social support networks, most studies have centered on community samples. It is important to also examine SITB disclosure in clinical samples as rates of disclosure, the recipients of these disclosures, and levels of support may vary in clinical samples. Addressing these gaps, this study focused on a clinical sample of adolescents, expanding on prior research primarily focused on community settings. Furthermore, the current study examined the rates of disclosure of NSSI, suicide ideation, and suicide attempts and identified the individuals to whom they disclosed their SITBs. Differences in reasons for living, including self-reported parent and peer support, self-acceptance, future optimism, and fear of suicide, were also examined for adolescents who did and did not disclose each SITB. It was hypothesized that rates of disclosure for NSSI and suicide ideation would be relatively high, at least around 70%, as previous research with community samples in the United States report these rates, and rates for a clinical sample may differ. Rates of disclosure for suicide attempts were expected to be higher than for NSSI and suicide ideation, based on results from previous research showing that more severe SITBs are disclosed at higher rates. It was also hypothesized that adolescents who did disclose their DITB would report more parent and peer support compared to those who did not disclose their self-harm.

## Method

### Participants

Data were collected from 100 adolescents recruited from a children’s crisis stabilization unit in the south-central region of the United States as part of a study on factors relating to suicide ideation. Adolescents were admitted to the crisis stabilization unit for a range of behavioral issues not limited to SITBs. The mean age of the sample was 14.61 (SD = 1.52). The majority of the sample (79%) identified as White, 4% as Black, 8% as multi-ethnic, 5.2% as Hispanic/Latinx, and 4% identified as another ethnicity (Asian, Hawaiian Native, Middle Eastern and North African). The majority identified as cisgender girls (67.5%), 28.6% as cisgender boys, 1.3% as transgender, and 2.6% as “not sure.” In terms of sexual orientation, 66.7% identified as heterosexual, 27.7% as bisexual/pansexual, and 5.6% as gay/lesbian. Inclusion criteria were that participants must be between 12 and 17 years old, admitted as patients in the children’s crisis stabilization unit, and proficient in English.

### Measures

#### Self-Harm Behavior Questionnaire (SHBQ).

The Self-Harm Behavior Questionnaire (SHBQ; Gutierrez et al., 2000) is a self-report measure that assesses self-harm behavior history in four different domains: suicide ideation, suicide threats, NSSI, and suicide attempts. The current study focused on three domains: Suicide Ideation (“Have you ever talked or thought about wanting to die?”), Suicide Attempt (“Have you ever attempted suicide?”), and NSSI (“Have you ever hurt yourself on purpose? [e.g., scratched yourself with fingernails or sharp object]”). If participants endorse history of suicide ideation, suicide attempts, or NSSI, they are directed to follow-up questions that assess for characteristics such as intent, frequency, duration, methods, preparation, and need for medical attention, depending on the self-harm behavior. There is a follow-up question for each behavior that asks about disclosure. For NSSI the follow-up question is “Have you ever told anyone that you had done these things?” If they answer yes, another follow-up question asks, “If yes, who did you tell?” and participants write in their responses. For suicide attempts, the follow-up question is “Did you tell anyone about the attempt?” which is followed up with a question prompting them to write in to whom they disclosed. For suicide ideation, the follow-up question is “With whom did you discuss this?” and space to write in a response. In the current study, adolescents were identified as having NSSI, suicide ideation, and suicide attempt history by their responses to the first item in each domain (yes/no). Among adolescents who reported history of the respective self-harm behavior, items regarding disclosure were used to identify rates of disclosure, and qualitative responses about to whom they disclosed were coded and categorized. Responses were coded in two different ways; once to identify disclosure to parents vs. others, and once to identify disclosure to peers vs. others. For parent coding, if adolescents listed more than one person to whom they disclosed, if at least one person listed was a parent, they were coded as disclosing to parents. If none of the people listed was a parent, they were coded as disclosing to others. For peer coding, if adolescents listed more than one person to whom they disclosed, if at least one person was a peer, they were coded as disclosing to peers. If none of the people listed was a peer, they were coded as disclosing to others. All SHBQ responses were coded by two independent, trained research assistants. Discrepancies were minimal (less than 5%) and were resolved by the principal investigator. Quantitative and qualitative responses on the SHBQ are scored and weighted for severity (Gutierrez & Osman, 2008). Total scores are calculated for each domain, and the four domains are summed for an overall total score. Higher domain and total scores indicate greater self-harm history and severity. The SHBQ has displayed strong internal reliability and convergent validity with other suicidal ideation and behavior measures in adolescent samples (α = 0.89–0.96; Gutierrez et al., 2000). Internal consistency was good in the current study for the SHBQ total score (α = 0.89).

#### Reasons for Living Inventory for Adolescents (RFL-A)

The Reasons for Living Inventory for Adolescents (RFL-A; Osman et al., 1998) is a 32-item measure that assesses the importance of various reasons for not considering suicide on a 6-point Likert scale ranging from 1 (*not at all important*) to 6 (*extremely importan*t). The RFL-A items can be grouped into five subscales: Future Optimism, Peer-Acceptance and Support, Family Alliance, Self-Acceptance, and Suicide-Related Concerns. Although these scales indirectly assess constructs like family and peer support, future optimism, etc., items are similar to those on the Multidimensional Scale for Perceived Social Support. RFL-A subscales also show moderate to strong correlations with concurrent measures in adolescent samples, respectively (Gutierrez et al., 2000; Zimet et al., 1988). For example, the family alliance subscale is negatively correlated with other measures of family problems, and the peer acceptance and support subscale are negatively correlated with other measures of alienation (Gutierrez et al., 2000). Subscale scores are calculated by taking the mean of the items, and the total score is the mean of all items, with all scores ranging from 1–6. The RFL-A has demonstrated strong internal consistency (α = 0.89–0.97) and test–retest reliability (α = 0.70) for the total score, and the Peer-Acceptance and Support and Family Alliance subscale also shows strong internal consistency in adolescent samples (α = 0.89–0.92; α = 0.93–0.95; Gutierrez et al., 2000; Osman et al., 1998). In the current study, all subscales exhibited excellent reliability (α = 0.94—0.96).

### Procedure

The study received full approval from the Institutional Review Board at Western Kentucky University. Upon admission to the children’s crisis stabilization unit, informed consent information was provided to the parent or guardian within the admission paperwork. Parent consent forms indicated that adolescents would be approached to participate in the study during their stay at the crisis unit. Inclusion criteria were to be between the ages 12–17, able to complete the study in English, and not currently intoxicated or experiencing psychosis symptoms. The average length of stay for the crisis unit is seven days, and most adolescents were recruited into the study around the midpoint of their stay. Members of the research team made regular visits to the unit (on average, weekly), to recruit adolescents with positive parent consent. Adolescents were given information about the study and asked to sign assent forms if they wished to participate. Research team members then met with adolescents in private rooms to complete the research protocol, which began with the University of Washington Risk Assessment Protocol (UWRAP; Linehan, 2014) to assess baseline levels of distress and self-harm urges. Research assistants then guided adolescents through the self-report measures and the UWRAP was administered again at the end of the protocol to assess any changes in distress or self-harm urges. Adolescents who reported increased changes were noted and reported to staff clinicians for follow-up. Upon completion of the study, participants were thanked for their time and provided with a $20 gift card which they received at discharge.

### Data Analytic Plan

Sample size was a product of the recruitment target of the parent study from which the data came. All variables were examined for missing data. All 100 participants responded to questions about NSSI and suicide attempt history, and 98 participants responded to the question about suicide ideation history. Complete data were available from all but 1–2 participants on each of the RFL-A subscales; 1 missing on Family Alliance, Self-Acceptance, and Future Optimism, and 2 missing on Suicide-Related Concerns and Peer Acceptance. Given the small amount of missing data, analyses were run for participants with complete data. Frequencies were run to determine rates of NSSI, suicide ideation, and suicide attempt history, and rates of disclosure for each SITB. Among those endorsing each type of SITB, frequencies were run to determine if they had disclosed the behavior to anyone (yes/no), and to whom they had disclosed (parents vs. others and peers vs. others). To compare RFL-A subscale scores between SITB disclosure groups, a series of ANOVA were used. First, adolescents who disclosed NSSI were compared to those who did not disclose NSSI on all RFL-A subscales. Next, adolescents who reported disclosing NSSI to parents were compared to those who disclosed NSSI to others (other family members, friends, therapists, etc.) on all RFL-A subscales. Lastly, adolescents who reported disclosing to peers were compared to those who disclosed NSSI to others (family members, therapists, etc.). For adolescents with SI history, an ANOVA was run to compare those who disclosed their SI to those who did not disclose their SI. Another ANOVA was used to compare those who disclosed SI to parents to those who disclosed to others. Lastly, adolescents who reported disclosing to peers were compared to those who disclosed SI to others (family members, therapists, etc.). Finally, an ANOVA was run to compare adolescents who disclosed a suicide attempt to parents to those who disclosed an attempt to others, and to compare adolescents who disclosed a suicide attempt to peers to those who disclosed an attempt to others. Eta-squared (ƞ^2^) values were computed for effect sizes; small effect sizes are > 0.01- and < . 06, medium effect sizes are between > 0.06 and < 0.14, and large effect sizes are > 0.14 (Cohen, 1988). Comparisons between disclosure and non-disclosure of suicide attempts was not possible since all but two adolescents who completed the disclosure questions reported disclosing to at least one person.

## Results

### Rates of Self-Harm Behavior and Disclosure

Within the sample, 70% reported lifetime NSSI (*n* = 70). Of those with NSSI history, 77.1% reported that they had disclosed the NSSI (*n* = 54), 21.5% reported that they had not disclosed NSSI (*n* = 15), and one participant did not answer this question (1.4%). For suicide ideation, 77% reported lifetime history (*n* = 76). Among those with suicide ideation history, 75% reported that they had disclosed these thoughts to at least one person (*n* = 57), 15.8% reported they had not disclosed their SI *(n* = 12), and 9.2% did not respond to this question (*n* = 7). For suicide attempts, 49% reported lifetime history of at least one attempt (*n* = 49). Among those with suicide attempt history, 83.7% reported disclosing it (*n* = 41), 4.1% reported disclosing to no one (*n* = 2), and 12.2% did not answer this question (*n* = 6).

Overlap of SITBs were also examined. Within the sample of 100, six reported only NSSI, 12 reported only SI, and three reported only SA. Ten participants reported none of these SITBs. In terms of overlap, 23 reported both NSSI and SI history, five reported NSSI and SA history, five reported SI and SA history, and 36 reported histories of all three SITBs (NSSI + SI + SA).

Overlap of disclosure for SITBs was also examined. For the 36 participants who reported all three SITBs, chi-square analyses were used to examine if likelihood of one SITB disclosure was associated with likelihood of disclosure of another SITB. There were no significant differences when comparing SA to NSSI disclosure (χ^2^(1) = 1.91, *p* = 0.167), SA to SI disclosure (χ^2^(1) = 0.33, *p* = 0.566), and NSSI to SI (χ^2^(1) = 0.17, *p* = 0.681).

### To Whom Self-Harm Behavior is Disclosed

For adolescents who reported disclosing NSSI, 13.3% reported disclosing NSSI to parents only, 35.6% reported disclosing NSSI to parents and others, and 51.1% reported disclosing NSSI only to others. Adolescents who noted disclosing NSSI to parents only and to both parents and others were combined into one group. Therefore, reports of disclosure were almost evenly distributed between parents + others (48.9%) and others only (51.1%). The other category included close friends, therapists, and other family members. When examining peer disclosure groups, 9% reported disclosing to peers only, 15.5% reported disclosing to both peers and others, and 75.5% reported disclosing to others only. Therefore, reports of disclosure were not as evenly distributed between peers + others (24.5%) and others only (75.5%).

For adolescents who reported disclosing suicide ideation, 7% reported disclosing SI only to parents, 21% reported disclosing SI to parents and others, and 72% reported disclosing only to others. Adolescents who noted disclosing SI to parents only and to both parents and others were combined into one group. Therefore, 26.4% reported disclosing to parents + others, and 73.6% reported disclosing to others only. Similar to NSSI, the other category included family members, friends, and therapists. When examining peer disclosure groups, 37% reported disclosing to peers only, 17.4% reported disclosing to both peers and others, and 45.6% reported disclosing to others only. Therefore, reports of disclosure were almost evenly distributed between peers + others (54.4%) and others only (45.6%).

For adolescents who reported disclosing suicide attempts, 7.4% noted that only parents knew about it, 46.3% reported that both parents and others knew, 46.3% reported that only others knew about the attempt. Therefore, 53.7% reported that parents + others knew about it, and 46.3% reported that only others knew (e.g., other family members, friends, and therapists). When examining peer disclosure groups, 11% reported disclosing to peers only, 26% reported disclosing to both peers and others, and 63% reported disclosing to others only. Therefore, reports of disclosure were not as evenly distributed between peers + others (37%) and others only (63%).

### Differences in Reasons for Living by Disclosure Status

For adolescents with NSSI history, ANOVA was used to compare 1) those who disclosed and did not disclose their NSSI, 2) those who disclosed to parents compared to others, and 3) those who disclosed to peers compared to others. Adolescents who disclosed NSSI had significantly higher scores on the self-acceptance and future optimism subscales of the RFL-A compared to those who had not disclosed their NSSI (see Table 1). Effect sizes for both significant subscales were medium; 0.061 for self-acceptance and 0.065 for future optimism. No differences were found on RFL-A subscale scores when comparing adolescents who disclosed NSSI to parents and those who disclosed to others (see Table 1). However, when comparing adolescents who disclosed NSSI to peers and those who disclosed to others, those disclosing to peers had significantly lower scores on the suicide-related concerns subscale of the RFL-A, indicating less fear of death and suicide, with a medium effect size (0.102; see Table 1).

**Table 1:** Results for Comparisons of NSSI Disclosure on Reasons for Living Subscales

	NSSI Disclosure Yes (*n* = 53)	NSSI Disclosure No (*n* = 15)	*F* (1,66)	ƞ^2^	NSSI Disclosure Parents (*n* = 21)	NSSI Disclosure Others (*n* = 23)	*F* (1, 42)	ƞ ^2^	NSSI Disclosure Peers (*n* = 11)	NSSI Disclosure Others (*n *= 33)	*F* (1, 42)	ƞ ^2^
Family Alliance	4.09 (1.60)	3.72 (1.37)	0.67	.010	4.00 (1.44)	3.95 (1.85)	0.10	< .001	3.83 (1.57)	4.02 (1.69)	0.11	.003
Suicide-Related Concerns	3.29 (1.55)	3.19 (1.83)	0.04	.001	2.96 (1.33)	2.96 (1.69)	< 0.01	< .001	2.14 (1.04)	3.24 (1.55)	4.77*	.102
Self-Acceptance	3.55 (1.59)	2.62 (1.32)	4.26*	.061	3.27 (1.36)	3.20 (1.75)	.02	< .001	2.69 (1.56)	3.41 (1.54)	1.76	.041
Peer Acceptance	4.30 (1.46)	3.84 (1.90)	1.00	.015	3.99 (1.46)	4.67 (1.38)	2.52	.057	4.59 (1.45)	4.27 (1.46)	0.41	.010
Future Optimism	4.67 (1.13)	3.90 (1.59)	4.59*	.065	4.37 (1.27)	4.47 (1.20)	0.07	.002	4.06 (1.09)	4.55 (1.26)	1.28	.030

Subscales were from the Reasons for Living Inventory for Adolescents (RFL-A)

*p < .05

For adolescents with suicide ideation history, ANOVA was used to compare 1) those who disclosed and did not disclose their SI, 2) those who disclosed to parents compared to others, and 3) those who disclosed to peers compared to others. Adolescents who disclosed their suicide ideation had significantly higher scores on the family alliance subscale of the RFL-A compared to those who had not disclosed. The effect size for this significant mean difference was medium (0.072). There were no significant differences found for RFL-A subscale scores when comparing adolescents who disclosed to parents compared to those who disclosed to others (see Table 2). However, when comparing adolescents who disclosed to peers to those who disclosed to others, there was a difference in the suicide-related concerns subscale of the RFL-A that approached significance and showed a medium effect size (*p* = 0.069; ƞ^2^ = 0.06). The results followed the same pattern as was found for NSSI, with those disclosing to peers showing less fear of death and suicide compared to those disclosing to others (see Table 2).

**Table 2:** Results for Comparisons of Suicide Ideation Disclosure on Reasons for Living Subscales

	SI Disclosure Yes (*n *= 57)	SI Disclosure No (*n* = 12)	*F* (1, 67)	ƞ ^2^	SI Disclosure Parents (*n* = 15)	SI Disclosure Others (*n* = 41)	*F* (1, 54)	ƞ ^2^	SI Disclosure Peers (*n* = 30)	SI Disclosure Others (*n* = 26)	*F* (1, 54)	ƞ ^2^
Family Alliance	4.19 (1.45)	3.11 (1.66)	5.23**	.072	4.60 (1.06)	4.07 (1.85)	1.47	.027	3.93 (1.41)	4.53 (1.46)	2.43	.043
Suicide-Related Concerns	3.41 (1.68)	3.83 (1.29)	0.68	.010	3.46 (1.47)	3.44 (1.75)	< 0.01	< .001	3.07 (1.74)	3.88 (1.50)	3.45*	.060
Self-Acceptance	3.37 (1.60)	3.47 (1.25)	0.05	.001	3.18 (1.53)	3.48 (1.64)	0.38	.007	3.15 (1.63)	3.68 (1.55)	1.54	.028
Peer Acceptance	4.27 (1.49)	4.17 (1.87)	0.05	.001	4.29 (1.52)	4.32 (1.48)	< 0.01	< .001	4.52 (1.37)	4.06 (1.58)	1.35	.024
Future Optimism	4.59 (1.27)	4.49 (1.23)	0.06	.001	4.69 (1.19)	4.64 (1.19)	0.02	< .001	4.47 (1.28)	4.86 (1.04)	1.46	.026

Subscales were from the Reasons for Living Inventory for Adolescents (RFL-A)

*p < .10, ** p < .05

For adolescents with suicide attempt history, ANOVA was used to compare 1) those who disclosed to parents compared to others, and 2) those who disclosed to peers compared to others. Since only 4% reported not disclosing their attempt to anyone, comparisons could not be made for those who did or did not disclose. There were no significant differences found on RFL-A subscale scores for adolescents who disclosed to parents compared to those who disclosed to others. There was a significant difference on the self-acceptance subscale of the RFL-A when comparing those who disclosed to peers to those who disclosed to others, with the peer disclosure group showing lower scores than the other group and a medium effect size (0.11; see Table 3).

**Table 3:** Results for Comparisons of Suicide Attempt Disclosure on Reasons for Living Subscales

	SA Disclosure Parents (*n* = 22)	SA Disclosure Others (*n *= 18)	*F* (1, 38)	ƞ ^2^	SA Disclosure Peers (*n *= 13)	SA Disclosure Others (*n* = 27)	*F* (1, 38)	ƞ ^2^
Family Alliance	3.88 (1.75)	4.27 (1.38)	0.60	.016	4.07 (1.56)	4.04 (1.62)	.004	< .001
Suicide-Related Concerns	2.98 (1.43)	3.4 (1.61)	0.76	.020	2.64 (1.60)	3.39 (1.43)	2.16	.055
Self-Acceptance	3.01(1.32)	3.35 (1.81)	0.48	.013	2.44 (1.33)	3.51 (1.54)	4.64*	.109
Peer Acceptance	4.34 (1.65)	4.42 (1.61)	0.02	.001	4.91 (1.45)	4.11 (1.65)	2.18	.054
Future Optimism	4.35 (1.35)	4.56 (1.29)	0.25	.006	4.24 (1.34)	4.54 (1.27)	0.46	.012

Subscales were from the Reasons for Living Inventory for Adolescents (RFL-A)

*p < .05

## Discussion

The current study examined the rates of disclosure of nonsuicidal self-injury (NSSI), suicide ideation, and suicide attempts in a clinical sample of adolescents, and identified the individuals to whom they disclosed their SITBs. Based on prior research, it was anticipated that disclosure of NSSI and suicide ideation would be around 70% and that rates of disclosure for suicide attempts would be even higher. Instead, the findings revealed higher disclosure rates across all types of SITBs (NSSI, suicide ideation, and suicide attempts). The highest disclosure rates were reported for suicide attempts (83.7%), followed by NSSI (77.1%), and suicide ideation (75%). This is contrary to the results in the Fox et al. (2022) study in which adolescents with history of mental health treatment and SITBS more frequently disclosed NSSI compared to suicide ideation or attempts. One reason for differences in disclosure rates may be due to the clinical nature of our sample. Although adolescents were admitted to the crisis stabilization unit for a wide range of behavioral issues (not limited to SITBs), a parent or guardian must accompany the child at the time of admission. Considering this, if the adolescent were admitted for SITB-related concerns, the parent or guardian would likely have already known about the behavior. Additionally, since enrollment into the study occurred at any time during the adolescent’s stay, they may have had coaching/therapy with a counselor about how to deal with SITBs, which may involve telling a trusted adult. While previous research indicates that adolescents generally prefer disclosing SITBs to peers (Demuthova et al., 2020; Eskin, 2003; Fox et al., 2022), the data from the current study revealed that adolescents were equally likely to disclose NSSI and suicide attempts to parents and peers but were more likely to disclose suicide ideation to others compared to parents. Again, these divergent findings may be influenced by the clinical setting from which our participants were recruited, since it may have been disclosure of direct self-harm behaviors such as NSSI and suicide attempts that contributed to their admission to the crisis unit. However, some adolescents in the sample still reported self-harm thoughts and behaviors that they had not disclosed to anyone.

Although the results of the current study revealed slightly higher disclosure rates compared to previous studies, the current study also aimed to explore the characteristics of these disclosures. It was initially hypothesized that adolescents who disclosed SITBs (to anyone) would report greater support from parents and peers compared to non-disclosers. However, this was only significant for adolescents who disclosed suicide ideation, particularly in terms of family support rather than peer support. Adolescents who perceive their family as supportive may be more inclined to disclosure their suicide ideation to anyone, considering that fear of family finding out may not be a barrier. Additionally, recent research has also found that self-harm disclosure to parents has been perceived as more helpful and more likely to lead to receiving professional help (Whitlock et al., 2015). This finding is consistent with previous research indicating that parent support may buffer the impact of SITBs in adolescence (Connor & Rueter, 2006; Kidd et al., 2006). However, adolescents may not have similar experiences with their peers. Hasking et al. (2015) examined characteristics of NSSI disclosure in a sample of Australian adolescents and found that those who had disclosed their behaviors to a peer reported a decrease in perceived social support over time. This finding, combined with our own, could indicate that adolescents may be experiencing less helpful reactions from their peers after the disclosure of SITBs.

When examining characteristics of adolescents who did or did not disclose their SITBs, a few patterns emerged from the data. First, across all SITBs, there were no differences in the reasons for living subscales (i.e., family alliance, peer-acceptance and support, self-acceptance, future optimism, and suicide-related concerns) between adolescents who disclosed to parents and adolescents who disclosed to others (e.g., peers, therapists, other family members, etc.). One potential reason for this finding may again be related to the clinical nature of the study. Parent disclosure in the current study is likely to be higher compared to a community sample considering a parent or guardian must be with the adolescent at time of admission to the crisis stabilization unit. For this reason, parent disclosure may have been inflated, limiting our ability to make comparisons between adolescents disclosing to peers and/or others.

On the other hand, adolescents who disclosed a suicide attempt to peers had lower self-acceptance compared to those who disclosed to others (e.g., parents, therapists, etc.). Given the cross-sectional nature of the data, it is uncertain in which direction this association occurs. One possibility is that adolescents who disclosed their suicide attempt to peers perceived it to be a negative or unhelpful experience, which may have led to decreased self-acceptance. Hasking et al. (2015) found similar results as adolescents in that study reported decreased social support over time after disclosing NSSI to a peer. Taken together, these findings could indicate that adolescents may be having negative experiences disclosing their SITBs to peers. Another possible explanation for this finding is that adolescents with lower self-acceptance may be more likely to disclose their suicide attempt to a peer as a way of seeking validation and support. The Interpersonal Theory of Suicide (Joiner, 2005) highlights the influence of thwarted belongingness and perceived burdensomeness as risk factors for suicide ideation, both experiences that can be influenced by perceived social support (i.e., parents and peers). Adolescents who experience desire for suicide (thwarted belongingness + perceived burdensomeness) and have made a suicide attempt may be disclosing to their peers as a way to fill a void in their social relationships.

Lastly, adolescents who disclosed NSSI and suicide ideation to peers reported lower fear of death and suicide compared to those who disclosed to others (e.g., parents, therapists, etc.). One reason for this finding may be explained by the clinical nature of our study. Although not all participants were admitted into the crisis stabilization for SITB related reasons, adolescents may view the unit as a safe space and be more inclined to share their SITB experiences with their peers. However, peers with similar experiences in these settings might impact other patients by normalizing these behaviors and possibly reducing fear of suicide/death. Kerr et al. (2006) examined the relationship between social support and adolescent suicide ideation in a clinical setting and found that for males, peer support associated with an increase in suicide ideation. Similarly, Taiminen et al. (1998) examined NSSI contagion among adolescents on an inpatient unit where contagion was defined as two or more acts of NSSI that involved two or more patients and occurred on the same or consecutive days. Results showed that contagion may have been a factor in NSSI episodes among adolescents on an inpatient unit, specifically in girls with depressive symptoms who used cutting as an NSSI method (Taiminen et al., 1998). Considering previous research and the current study findings, clinical settings may consider protocols that limit detailed disclosure and discussion about SITBs among adolescents, similar to protocols used for DBT Skills groups (Linehan, 2014). These results could also suggest that adolescents who reported NSSI to peers had a lower fear of death and greater acquired capability for suicide. Disclosure of NSSI to parents or other professionals is more likely to lead to immediate intervention; thus, adolescents with a higher fear of their behaviors escalating to suicide or death, or those with more severe NSSI, might be more likely to disclose to these individuals for help-seeking purposes. Pisani and colleagues (2012) found that most adolescents who disclosed to adults did so to seek help, indicating they had a greater fear and less acceptance of their self-injurious behaviors. Additionally, adolescents might experience a greater fear of death or suicide after disclosing their NSSI to parents or therapists, who might respond more urgently or reactively than peers (Rissanen et al., 2009b).

## Limitations and Future Research

Although this study adds to the self-harm disclosure literature by examining characteristics of disclosure in a clinical sample of adolescents, it had a few limitations. First, the sample showed a lack of racial and ethnic diversity. Adolescents with minoritized racial or ethnic identities may have different support systems and beliefs about mental health that may look different in terms of disclosure rates, limiting generalizability of our results. Second, since only 4% reported not disclosing their suicide attempt, characteristics of disclosure could not be examined in depth. Additionally, the small sample size of the study limited the ability to detect significant differences between disclosure groups, potentially contributing to the null findings and further limiting generalizability. Third, adolescents were admitted to the crisis stabilization unit for various behavioral issues, including SITBs and behaviors considered endangering to others. Reasons for the adolescents’ admission to the unit were not recorded. This could pose a limitation as admission to the unit could be attributed to disclosure of SITBs, and, in turn, results may have been impacted by how adolescents in a clinical setting report disclosure experience. Fourth, the sample size and overlap of participants endorsing multiple SITBs led to some participants being included in multiple analyses for NSSI, suicide ideation, and suicide attempts. Similarly, in ANOVA analyses examining differences in reasons for living subscale scores between adolescent disclosure recipients, overlap among participants occurred because adolescents sometimes endorsed multiple disclosure recipients. Examining adolescents who endorsed multiple SITBs and multiple recipients of disclosure hindered direct comparisons of SITB disclosure to parents or peers. Fifth, the RFL-A assesses reasons for not considering suicide, and it is possible that this could have impacted responses on the RFL-A subscales for participants with no history of suicide ideation. Sixth, while this study identified the most cited recipients of disclosure for each SITB, the binary nature of the disclosure item on the SITB assessment measure restricted its ability to capture recipients of individual disclosure events, posing a limitation. It should be noted that the analyses for the current study were correlational, and no causal interpretations can be drawn from the results. Significant differences observed in reasons for living subscale scores may reflect individual differences that influence the disclosure process or the choice of disclosure recipients. Conversely, it is possible that the disclosure of a SITB or the choice of disclosure recipient could also influence the significant differences found in certain subscale scores. These associations may also operate in a bidirectional manner. Future research should aim for a larger and more diverse sample and examine what happens after disclosure or if adolescents receive proper care upon disclosure.

## Conclusion

Adolescents who are in inpatient settings may have higher rates of disclosure for self-injurious thoughts and behaviors. However, not all adolescents in a crisis unit who report self-harm history have disclosed those experiences. Adolescents in clinical settings may experience higher rates of disclosure of SITBs, especially to parents. Previous research and the current findings indicate that adolescents may have less positive experiences disclosing SITBs to peers, which may also associate with lower self-acceptance. Staff in clinical settings may need to implement protocols limiting detailed discussions about SITBs in group settings, as previous research has reported negative outcomes (i.e., increased suicide ideation among boys, contagion of NSSI episodes among girls, and decreased fear of death). By examining the characteristics of disclosure of self-harm behaviors in adolescents, we can promote safe and comfortable environments to facilitate these conversations and implement self-harm prevention.

## Data Availability

Data used in this study are available by request.
